# Quantity and Quality of Healthcare Professionals, Transfer Delay and In-hospital Mortality Among ST-Segment Elevation Myocardial Infarction: A Mixed-Method Cross-Sectional Study of 89 Emergency Medical Stations in China

**DOI:** 10.3389/fpubh.2021.812355

**Published:** 2022-01-24

**Authors:** Qiang Zhou, Wenya Tian, Rengyu Wu, Chongzhen Qin, Hongjuan Zhang, Haiyan Zhang, Shuduo Zhou, Siwen Li, Yinzi Jin, Zhi-Jie Zheng

**Affiliations:** ^1^Shenzhen Center for Prehospital Care, Shenzhen, China; ^2^Department of Chronic Disease Epidemiology, School of Public Health, Yale University, New Haven, CT, United States; ^3^Department of Global Health, School of Public Health, Peking University, Beijing, China; ^4^Institute for Global Health, Peking University, Beijing, China

**Keywords:** STEMI, healthcare professional, transfer delay, in-hospital mortality, mixed methods

## Abstract

**Background:**

Transfer delay provokes prolongation of prehospital time, which contributes to treatment delay that endangers patients with ST-segment elevation myocardial infarction (STEMI). A key constraint in reducing transfer delay is the shortage of emergency healthcare workers. This study was to explore the influence of the quality and quantity of healthcare professionals at emergency medical stations on transfer delay and in-hospital mortality among STEMI patients.

**Methods:**

A cross-sectional study using mixed methods was conducted at 89 emergency stations in 9 districts in China's Shenzhen province. Based on a sample of 31 hospitals, 1,255 healthcare professionals, and 3,131 patients with STEMI, a generalized linear model was used to explore the associations between the quality and quantity of healthcare professionals and transfer delay and in-hospital mortality among STEMI patients. Qualitative data were collected and analyzed to explore the reasons for the lack of qualified healthcare professionals at emergency medical stations.

**Results:**

The analysis of the quantity of healthcare professionals showed that an increase of one physician per 100,000 individuals was associated with decreased transfer delay for patients with STEMI by 5.087 min (95% CI −6.722, −3.452; *P* < 0.001). An increase of one nurse per 100,000 individuals was associated with decreased transfer delay by 1.471 min (95% CI −2.943, 0.002; P=0.050). Analysis of the quality of healthcare professionals showed that an increase of one physician with an undergraduate degree per 100,000 individuals was associated with decreased transfer delay for patients with STEMI by 8.508 min (95% CI −10.457, −6.558; *P* < 0.001). An increase of one nurse with an undergraduate degree per 100,000 individuals was associated with decreased transfer delay by 6.645 min (95% CI −8.218, −5.072; *P* < 0.001). Qualitative analysis illustrated that the main reasons for low satisfaction of healthcare professionals at emergency medical stations included low income, limited promotion opportunities, and poor working environment.

**Conclusions:**

The quantity and quality of emergency healthcare professionals are key factors influencing transfer delay in STEMI patients. The government should increase the quantity of healthcare professionals at emergency medical stations, strengthen the training, and improve their performance by linking with clinical pathways to enhance job enthusiasm among emergency healthcare professionals.

## Introduction

ST-segment elevation myocardial infarction (STEMI) is a severe subtype of coronary heart disease and is a significant cause of mortality worldwide. The hospitalization rate of patients with STEMI in China increased nearly four-fold between 2001 and 2011. The rate of STEMI among male patients increased from 4.6 to 18 per 100,000 individuals (1). Most STEMI-related deaths occur in the first few hours of disease manifestation, with 40–65% occurring in the first hour (2). It has also been shown that every 30 min delay in reperfusion reduces a patient's life expectancy by 1 year (3). Therefore, the duration of time from symptom onset to reperfusion therapy, which includes patient delay, transfer delay, and in-hospital delay, is a crucial factor determining mortality in STEMI patients. The primary cause of delay in reperfusion is system delay (4), which mainly comprises transfer delay. Compared with door-in-door-out patients, the door-to-balloon time for patients who are directly transferred to emergency medical stations with in-hospital percutaneous coronary intervention capabilities is shorter and their prognosis is better (5).

Emergency medical services (EMS) systems have been established since the 1950s and increasingly emerged as an important part of the health service delivery system in China. The EMS system in China includes pre-hospital emergency centers that provide pre-hospital care, and hospital emergency departments and intensive care units that provide in-hospital care (6). In China, the patterns of pre-hospital EMS include stand-alone type, dependent type, directive type and others. Ambulances can be directed to an emergency center (stand-alone type) or hospital (directive type), and EMS is provided by the emergency center (pre-hospital type) and hospital (dependent type). The pre-hospital EMS supported by Shenzhen in Guangdong Province of China, belongs to the directive type. The Shenzhen Emergency Medical Center is a command center for unified communications, and is responsible for coordinating EMS across the city (7). After receiving an emergency call, the emergency medical center dispatches an ambulance staffed with healthcare professionals from the nearest emergency medical station to the emergency scene according to the patient's condition. An ambulance has at least three staff members: a doctor, a nurse and a driver. Advanced life support equipment and 12-lead electrocardiograph (ECG) are standard equipment on board. This EMS system aims to facilitate effective use of medical resources, shorten response time, and improve rescue efficiency (8).

Shenzhen currently has a sufficient number of ambulances. All ambulances are well equipped with medical supplies including monitoring apparatus for the resuscitation of critically ill patients (9), such as electrocardiograph (ECG), ventilator, and cardiopulmonary resuscitation device. Sufficient healthcare professionals are needed to provide patients with available and sustainable healthcare services (10). The physician and nurse are responsible for pre-hospital diagnosis and treatment; they need to identify symptoms immediately, transmit ECG results to the hospital as soon as possible, and rapidly communicate with physicians in the hospital emergency department. Therefore, the quantity and quality of physicians and nurses affect pre-hospital emergency response time, which in turn influences clinical outcomes (11).

Most previous studies focused on the association between transfer modes (12), delay time, and mortality among patients with STEMI (3, 13–15). Although some researchers explored the role of general practitioners/primary healthcare physicians in treating patients with STEMI in remote areas (16), the influence of the quality and quantity of healthcare professionals on transfer delay and in-hospital mortality have received little attention. Currently, China is facing problems associated with scarcity and uneven distribution of healthcare professionals for pre-hospital EMS. A major constraint is the shortage of healthcare workers, which means it is not possible to increase the number of operating ambulances because the basic personnel allocation for ambulances cannot be met (17).

The purpose of the present study was to explore the influence of the quality and quantity of healthcare professionals at emergency medical stations on transfer delay and in-hospital mortality among patients with STEMI in China. The results of this study could provide an implication for the allocation of emergency healthcare professionals in developing countries with the similar EMS systems.

## Methods

### Data Collection and Patients

This cross-sectional study used regular report data on the allocation of healthcare resources at emergency medical stations and database on chest pain patients from hospitals. The patient database contained case data for patients with STEMI from 31 hospitals in nine districts of Shenzhen in 2019. The data reporting of each emergency station is based on a unified reporting platform with a standardized format and data elements (Appendix 1). After filling in the data, the EMS center checked it uniformly, and there are full-time data managers who returned the abnormal values or logically improper values to each station for re-checking. For analysis, we adopted a data double-entry approach to ensure accurate data entry. The regular report data at emergency medical stations comprised the number and individual characteristices for physicians and nurses at 89 emergency medical stations in nine districts of Shenzhen in 2019. Questionnaire for regular report data is shown in Appendix 2. The two databases were merged based on district and hospital coding, and patients were excluded if they were transferred to other hospitals. Finally, 31 hospitals, 1,255 healthcare professionals, and 3,131 patients with STEMI were included in our analyses.

## Quantitative Data

### Measurement

#### Independent Variables

The numbers of physicians and nurses per 100,000 individuals in each district of Shenzhen were used to measure the quantity of healthcare professionals. We used the number of physicians holding undergraduate degrees and nurses holding college degrees per 100,000 individuals to measure the quality of healthcare professionals.

#### Dependent Variables

We used transfer delay and in-hospital mortality of patient with STEMI as the dependent variables. Transfer delay referred to the total time from calling 120 to hospital admission for each patient with STEMI. In-hospital mortality was based on the discharge diagnosis (death or not).

#### covariates

Based on empirical studies, we controlled for variables that may act as potential confounders (18). The covariates used in this study were: patients' age, gender, hypertension or not, normal heart rate or not (normally between 60–100 bpm), clinical stage of heart failure caused by acute myocardial infarction (Killip class I–IV), and emergency risk stratification (low risk = 0, moderate risk = 1, and high risk = 1).

## Qualitative Data

A semi-structured interview was conducted to explore the reasons for the lack of quantity and quality of healthcare professionals at emergency medical stations. Interview guide is shown in Appendix 3. Two well-trained authors (WYT, RYW) were recruited for conducting interviews. All interviews were entirely voluntary. Two physicians, two nurses, and one director from each emergency medical station were selected, giving a total of 445 interviewees. This selection was based on occupation, gender, age, and educational attainment to ensure the interview results were representative of the responses of all healthcare professionals and directors in these emergency medical stations. The interview questions for physicians and nurses explored job enthusiasm, job satisfaction, and preferences for different incentive factors. Questions for directors included management of emergency medical dispatch, personnel incentive mechanism, and reasons for lack of healthcare professionals. Interviews were conducted in an individual meeting room without any non-participants for privacy and comfort of the interviewees. Each interview lasted for 30 min with on-spot notes and audio recording. The interviews ended when data saturation was reached.

Content analysis was applied to underpin the study. Themes were identified in advance, and the coding framework was divided into the individual-, the institutional- and the health system level factors influencing healthcare professionals' motivation and retainmance, with each of level sub-coded by workload, autonomy, incentives, working environment, and other specific motivators. Theoretical coding was developed based on the analysis consumption of this study using NVivo 12.0. Two well-trained authors (CZQ, HJZ) were recruited for verbatim transcriptions and coding of the audio records. Codes were revised and improved through reviewing transcripts and discussion.

## Statistical Analyses

We described the basic characteristics of patients with STEMI and the quantity and quality of physicians and nurses. The transfer delay and in-hospital mortality of patients with STEMI were descriptively analyzed using analysis of variance and chi-square tests (*P* < 0.05). Given the data type of the dependent variables, linear regressions were used to explore the association between the quality and quantity of healthcare professionals and Transfer delay among patients with STEMI, because the transfer delay time is a continuous variable, while logistic regressions were used to explore the association between healthcare professionals and in-hospital mortality among patients with STEMI because in-hospital mortality is a categorical variable. Independent variables including the numbers of physicians and nurses per 100,000 individuals in each district of Shenzhen and the number of physicians holding undergraduate degrees and nurses holding college degrees per 100,000 individuals were included in the regression (19). Covariates (patients' age, gender, hypertension or not, normal heart rate or not, clinical stage of heart failure caused by acute myocardial infarction, and emergency risk stratification) were included in all models, and a 95% confidence interval (CI) for the regression coefficient was provided. All statistical analyses were performed using Stata V.15.1 (Stata Corp., College Station, TX, USA).

## Results

### Participants' Characteristics

The mean age of the 3,131 patients with STEMI was 56.98 years, and 17.18% were women. Most patients had normal heart rate (75.98%), low emergency risk (77.93%), and were classified as Killip class I (78.25%) (Table 1).

**Table 1:** Patient-level characteristics of study participants.

	**N**	**%**
**Patient-level characteristics**		
Number of hospital admissions	3,131	-
Age (years)^*^	56.98	13.71
Female	538	17.18
Heart rate		
Normal	2,379	75.98
Arrhythmia	752	24.02
Emergency risk		
Low	2,440	77.93
Medium	380	12.14
High	311	9.93
Hypertension	1,463	46.73
Killip class		
I	2,450	78.25
II	181	5.78
III	54	1.72
IV	130	4.15

* *Mean (SD)*.

There were 4.444 physicians and 5.073 nurses per 100,000 individuals in Shenzhen. The highest physicians density was those with undergraduate degrees (3.238 per 100,000), which was far higher than the density of physicians with other degrees. The highest nurse density was observed for those with undergraduate degrees (2.525), followed by those with college degrees (2.229) (Table 2).

**Table 2:** District-level characteristics of health care professional.

	**Physicians mean (SD)**	**Nurses mean (SD)**
Number of health care professionals per 100,000 individuals	4.444 (4.348)	5.073 (5.771)
Number of health care professionals with graduate degrees per 100,000 individuals	0.516 (1.667)	0.023 (0.231)
Number of health care professionals with undergraduate degrees per 100,000 individuals	3.238 (1.638)	2.525 (4.206)
Number of health care professionals with junior college degrees per 100,000 individuals	0.675 (0.397)	2.229 (3.515)
Number of health care professionals with technical secondary degrees per 100,000 individuals	0.053 (0.311)	0.311 (1.004)
Number of health care professionals with high school degrees per 100,000 individuals	0.023 (0.943)	0.008 (0.104)
Number of health care professionals with age under 25 per 100,000 individuals	0.045 (0.528)	0.811 (1.709)
Number of health care professionals with age between 25 and 34 per 100,000 individuals	1.327 (1.906)	3.117 (4.004)
Number of health care professionals with age between 35 and 44 per 100,000 individuals	1.926 (2.614)	1.046 (2.035)
Number of health care professionals with age over 45 per 100,000 individuals	1.160 (1.952)	0.121 (0.407)

Table 3 shows the transfer delay and in-hospital mortality for patients with STEMI in districts with differing densities of healthcare professionals. We divided the density of physicians and nurses equally into three levels by district (low, middle, and high). The least transfer delay for patients with STEMI was found in the three districts with high physician density (medians 38 min, 26 min, and 60 mins; *P* < 0.001). The greatest median transfer delay for patients with STEMI was found in the three districts with low nurse density (48 min, 32 min, and 70 min; *P* < 0.001).

**Table 3:** Healthcare professionals at district level and transfer delay and in-hospital mortality of STEMI patients.

	**Transfer delay, minutes (median, q1 q3)**	***P***	**In-hospital mortality, n (%)**	***P***
Total				
Districts with low physician density	46 (30, 72)	<0.001	71 (4.38%)	0.768
Districts with middle physician density	44 (32, 60)		39 (4.13%)	
Districts with high physician density	38 (26, 60)		24 (4.66%)	
Districts with low nurse density	48 (32, 70)	<0.001	26 (5.95%)	0.164
Districts with middle nurse density	40 (29, 56)		93 (4.08%)	
Districts with high nurse density	32 (24, 42)		15 (3.64%)	

### Association Between Quantity and Quality of Healthcare Professionals and Transfer Delay and In-hospital Mortality

The analysis of the quantity of healthcare professionals showed an increase of one physician per 100,000 individuals decreased transfer delay for patients with STEMI by 5.087 min (95% CI −6.722, −3.452; *P* < 0.001); an increase of one nurse per 100,000 individuals decreased this transfer delay by 1.471 min (95% CI −2.943, 0.002; *P*=0.050). Analysis of the influence of quality of healthcare professionals showed that an increase of one physician with an undergraduate degree per 100,000 individuals decreased the transfer delay for patients with STEMI by 8.508 min (95% CI −10.457, −6.558; *P* < 0.001); an increase of one nurse with an undergraduate degree per 100,000 individuals decreased this transfer delay by 6.645 min (95% CI −8.218, −5.072; *P* < 0.001) (Table 4).

**Table 4:** Associations between district-level healthcare professionals and patient-level transfer delay and in-hospital mortality.

	**Transfer delay (minutes)**			**In-hospital mortality (%)**		
	**Coeffect**	**95% CI**	***P***	**Coeffect**	**95% CI**	***P***
**Physicians**						
Number of physicians per 100,000 individuals	−5.087	[−6.722, −3.452]	<0.001	0.001	[−0.007, 0.008]	0.930
Number of physicians with undergraduate degrees per 100,000 individuals	−8.508	[−10.457, −6.558]	<0.001	−0.001	[−0.010, 0.008]	0.801
**Nurses**						
Number of nurses per 100,000 individuals	−1.471	[−2.943, 0.002]	0.050	0.003	[−0.004, 0.010]	0.385
Number of nurses with junior college degrees per 100,000 individuals	−6.645	[−8.218, −5.072]	<0.001	0.009	[0.001, 0.016]	0.022

### Reasons for Lack of Quantity and Quality of Healthcare Professionals

There was consistency between the data presented and the findings from interviews. In most cases, healthcare professionals at emergency medical stations had a low level of job satisfaction. Based on the themes identified in advance and the coding framework, the main reasons for this low satisfaction included low income, limited promotion opportunities, and poor working environment, which led to high job mobility and low attractiveness to highly educated professionals. Healthcare professionals indicated that their total income at emergency medical stations was lower than the average level for the city and did not match the high workload. Performance bonuses accounted for a low proportion of their total income. The salary gap between individuals with the same professional title was also relatively small, and did not reflect the difference in working competence.

The interviews with director from emergency medical stations found how the EMS delivery systems affected healthcare professionals' motivation and retainmance, which then transfer the influences on the lack of quantity and quality of healthcare professionals at emergency medical stations. The channels for promotion for healthcare professionals were limited. Unlike physicians and nurses in hospitals, those working at emergency medical stations had reached the ceiling for promotion if they held an intermediate professional title, regardless of their educational level and working competence. This mean that for healthcare professionals with the same advanced medical education, emergency medical stations were far less attractive than hospitals.

Working in an emergency center also requires night shifts, which demanded a high level of vigilance as emergency patients may need to be rescued at any time. Moreover, the family members of emergency patients were usually irritable, which was likely to intensify the doctor–patient relationship.

## Discussion

Current guidelines strongly recommend that patients presenting with STEMI symptoms seek EMS and arrive at the hospital by ambulance. EMS activation shortens the time to definitive treatment, facilitates the early acquisition of an ECG, allows for rapid activation by the treatment team, and significantly improves D2B times (20). In the United States, the most successful regional systems have implemented processes in which EMS providers obtain pre-hospital ECGs and activate cardiac catheterization labs prior to hospital arrival, bypass the emergency department (ED) when appropriate, and provide ongoing quality review and feedback (21). However, the results of a systematic review of emergency care in 59 low- and middle-income countries (LMICs) showed that mortality rates recorded in EDs in LMICs are many times higher than those commonly reported in high-income countries, and that most emergency providers in LMICs do not receive specialized training in emergency care (22). Previous studies have focused on the relationship between transfer patterns, delay time, and mortality in STEMI patients. However, to our knowledge, few studies have focused on the role and distribution of healthcare professionals in the EMS system. The present study took the lead to quantitatively explore the impact of quantity and quality of healthcare professionals at emergency medical stations, and pointed out that the health workforce is a key component of any EMS system and is critical in improving healthcare accessibility. Our study further qualitatively analyzed the reasons for the lack of quantity and quality of healthcare professionals, and raised strategies to increase coverage of the health workforce in EMS system are crucial to shortening response time and improving rescue efficiency.

First, our study demonstrated that the density of healthcare professionals was negatively associated with transfer delay for patients with STEMI. In China, each ambulance is usually equipped with a physician, a nurse and a driver (23). These professionals are responsible for providing EMS and treatment to patients during the transfer process, and monitoring patients before they are admitted to hospital (24). Our findings were consistent with previous studies, which showed that sufficient healthcare professionals are essential for providing basic health services and improving health outcomes (25, 26). High-quality healthcare professionals are widely recognized as the prerequisite for effective healthcare, and a critical factor that determines healthcare system performance (27). Pre-hospital EMS is an integral part of the healthcare system (28). In 2009, China initiated a comprehensive healthcare system reform, which included a goal of solving the lack of healthcare professionals. However, Shenzhen, as a first-tier city with a relatively high economic level, still faces a lack of emergency healthcare professionals. Such shortages may be particularly pronounced in emergency settings, where the work is demanding and salaries are often poor (22). Therefore, availability of healthcare professionals in EMS system should be improved to ensure the quality of pre-hospital emergency care.

Second, we found that a higher density of physicians with undergraduate degrees and a higher density of nurses with college degrees were associated with shorter transfer delay for patients with STEMI. For the emergency medical care of patients with STEMI, healthcare professionals are required to recognize the symptoms immediately, take ECG quickly, and rapidly transmit ECG results to the hospital emergency and cardiology departments, so that the hospital can complete the necessary preparations before patient arrival. This means the patient can bypass the emergency and cardiology departments after arriving at the hospital, and go directly to the catheterization laboratory for rapid treatment, thereby reducing transfer delay and in-hospital delay (29). The wrong decision by healthcare professionals that results in the patient not being sent to the catheterization laboratory in a timely manner can endanger the patient's health and even lead to death (30, 31). The professional competence of healthcare professionals is crucial in pre-hospital emergency medical care. A previous study showed that competent healthcare professionals are essential for providing healthcare services and determine the degree to which the services meet healthcare demand (32). To narrow the gap between supply and demand of emergency care, middle and long-term national plans for healthcare professionals in EMS system should be developed. First, appropriate incentive policies, including increase in incomes and opportunities in professional development, are needed for attracting and retaining qualified health workers in EMS system. Second, a higher education level and participation in education programs can also improve competence among healthcare professionals (33). However, the observation that most EDs in LMICs consist of providers without specialty training in emergency care, and most governments do not include emergency medicine in their medical education priorities, illustrates the opportunity to improve emergency care in LMICs (22). Therefore, the quality of healthcare professionals can be improved through on-the-job training, and skills in emergency care should be cultivated through medical education and skills training.

Third, although the quantity and quality of healthcare professionals are directly associated with transfer delay, they did not influence in-hospital mortality of patients with STEMI for multiple reasons. For example, the allocation of healthcare professionals at emergency medical stations mainly affects transfer delay, which accounts for approximately 25% of the total delay; in-hospital delay also affects treatment time and further influences in-hospital mortality. In addition, many factors influence the mortality of patients with STEMI, such as basic demographic characteristics (34), medical history (35), and delay caused by patient-level factors (36).

Fourth, our qualitative analysis revealed that the workload of these healthcare professionals was not proportional to their income, meaning incentive mechanisms failed to promote their motivation to work. The equalization of basic public health services (EBPH) policy implemented in 2009 expanded the coverage of public health services in China, and governments at all levels allocated funds for these services. However, the EBPH policy indicates special funds can only be used to support operating costs such as medical resource consumption and transportation, and cannot compensate for personnel expenses. Therefore, the input of healthcare professionals and the corresponding increase in workload do not receive sufficient rewards. The explanation for this phenomenon was consistent with previous studies; that is, healthcare workers are unwilling to provide medical services because they are concerned about the rapid increase in workload without corresponding financial returns (37–39). Furthermore, the performance of emergency medical stations is only evaluated by the number of ambulances dispatched, and not the quality of treatment. Thus, healthcare professionals may be motivated to drive the ambulances out, but not to provide treatment. There is also a growing trend not to transport patients in many Western European countries (40, 41). In addition, there are complex and multifactorial factors affecting the motivation for healthcare professionals in an ambulance not to treat patients. This decision is influenced by healthcare professionals, patients and their relatives, the healthcare system (referral or general physician), and auxiliary tools, such as a patient's refusal to accept therapy, disease severity of the patient, and the ability of healthcare professionals (34, 42), which results in prolonged transfer time. Therefore, the performance of emergency medical stations should be linked to service quality. As well as covering in-hospital mortality, service quality but should also cover clinical pathways, such as whether the healthcare professionals on the ambulance identified patient symptoms correctly, completed an ECG immediately, and transmitted the ECG results to the hospital emergency and cardiology departments in a timely manner.

This study has several inherent limitations. First, the main limitation is that it is a cross-sectional study, and our analyses are based on observational data that do not allow for causal inferences. This may lead to concerns about residual confounding or bias. Second, this study was limited to Shenzhen and the results may lack generalizability, so future studies should further explore data from other cities. Thirdly, the data were collected individually by 31 hospitals, which may introduce heterogeneity regarding the quality of data collection. Nevertheless, data reporting of each hospital and emergency station is based on the unified reporting platform with a standardized format and data elements.

## Conclusion

Ensuring sufficient quantity and quality of healthcare professionals at emergency medical stations is a top priority to reduce the delay in treatment of patients with STEMI. The government should increase the quantity of healthcare professionals at emergency medical stations, strengthen the training of professional personnel, and improve their performance by linking with clinical pathways to enhance job enthusiasm among emergency healthcare professionals.

## Data Availability Statement

The raw data supporting the conclusions of this article will be made available by the authors, without undue reservation.

## Ethics Statement

This project was approved by the Peking University Health Science Center Institutional Review Board (IRB00001052-21020). Informed consent was obtained from all participants prior to questionnaire administration. The patients/participants provided their written informed consent to participate in this study.

## Author Contributions

QZ and WT: drafting the manuscript, data analysis, and interpretation. RW: field investigation, data collection, and critical revision of article for important intellectual content. CQ, HoZ, HaZ, SL, and Z-JZ: critical revision of article for important intellectual content. SZ: data analysis and critical revision of article for important intellectual content. YJ: study concept and design, data analysis, and critical revision of article for important intellectual content. All authors gave final approval of the version to be published.

## Funding

This study is supported by Sanming Project of Medicine in Shenzhen (No. SZSM201911005), the National Natural Science Foundation of China (No. 71904004). The study sponsor has no role in study design, data analysis and interpretation of data, the writing of manuscript, or the decision to submit the paper for publication.

## Conflict of Interest

The authors declare that the research was conducted in the absence of any commercial or financial relationships that could be construed as a potential conflict of interest.

## Publisher's Note

All claims expressed in this article are solely those of the authors and do not necessarily represent those of their affiliated organizations, or those of the publisher, the editors and the reviewers. Any product that may be evaluated in this article, or claim that may be made by its manufacturer, is not guaranteed or endorsed by the publisher.

## Abbreviations

STEMI: ST-segment Elevation Myocardial Infarction
EMS: Emergency Medical Services
ED: Emergency Department
ECG: Electrocardiograph
CI: Confidence Interval
EBPH: Equalization of Basic Public Health Services.
