# Supplementary material for: Quantity and Quality of Healthcare Professionals, Transfer Delay and In-hospital Mortality Among ST-Segment Elevation Myocardial Infarction: A Mixed-Method Cross-Sectional Study of 89 Emergency Medical Stations in China
Source: Front Public Health. 2022 Jan 24;9:812355. doi: 10.3389/fpubh.2021.812355 (PMC8818716; doi:10.3389/fpubh.2021.812355)
Supplement: Supplementary file 1 [file Table_1.DOC]

Appendix Table 1. The registry data elements

| Category | Data Elements |
| --- | --- |
| Patient demographics | Age, sex, race, marriage, education, employment, insurance status |
| Medical history and risk factors | Height, weight, hypertension, hyperglycemia, hyperlipidemia, current smoker, family history of heart disease, prior cardiac history, prior revascularization |
| Prehospital treatment | Location of onset, onset date/time, transfer status, arrival date/time, date/time of first medical contact, date/time of first ECG, bypass ED/CCU |
| Presenting features and evaluation | ECG findings, consciousness, heart rate, systolic/diastolic blood pressure, cardiogenic shock, heart failure, Killip class, troponin concentration, serum creatinine, mini-GRACE risk score, preliminary diagnosis |
| In-hospital medication | Antiplatelet agents (aspirin, Clopidogrel/tegrillo), anticoagulant agents (warfarin, unfractionated heparin, low molecular weight heparin, bivalirudin, fondaparinux), intensive statin, β-blockers |
| In-hospital reperfusion strategy | LVEF assessment, Time to sign informed consent, primary PCI date/time (Cath lab activation date/time, Cath lab arrival date/time, door-to-balloon time), thrombolytic date/time, rescue PCI date/time, CABG date/time, recanalization date/time, TIMI class |
| In-hospital outcomes | Heart failure, length of stay, total cost, clinic outcomes (discharge/death/transfer to other hospitals) |
| Hospital Discharge | Principal discharge diagnosis, discharge medicines (DAPT, ACEI/ARB, statin, β-blockers), discharge counseling (Smoking cessation counseling, weight control counseling, blood pressure lowering medications, lipid lowering medications, glucose lowering medications, antithrombotic, follow up scheduling) |

ECG, Electrocardiograph; ED, emergency department; CCU, coronary care unit; PCI, percutaneous coronary intervention; LVEF, left ventricular ejection fraction; CABG, coronary artery bypass graft; DAPT, dual antiplatelet therapy; ACEI, angiotensin-converting enzyme inhibitor; ARB, angiotensin receptor blocker.
