# Supplementary material for: Quantity and Quality of Healthcare Professionals, Transfer Delay and In-hospital Mortality Among ST-Segment Elevation Myocardial Infarction: A Mixed-Method Cross-Sectional Study of 89 Emergency Medical Stations in China
Source: Front Public Health. 2022 Jan 24;9:812355. doi: 10.3389/fpubh.2021.812355 (PMC8818716; doi:10.3389/fpubh.2021.812355)
Supplement: Supplementary file 2 [file Table_2.DOCX]

Questionnaire for regular report data of the Shenzhen Center for Prehospital Care

Sanming Project of Medicine in Shenzhen

Name of Emergency Medical Stations:

Address of Institution: Shenzhen City

District/County

Street/Township

Name: Phone Number:

Date: / /

**Questionnaire Purpose and Confidentiality Statement**

This questionnaire was jointly developed by the Shenzhen Center for Prehospital Care and the School of Public Health, Peking University. We will strictly keep the survey subjects confidential, and the data collected will be used only for academic research. All the information will not appear in any publication, nor will it be used for any commercial purpose or administrative evaluation. In order to ensure the scientificity and effectiveness of the survey, please fill in truthfully. Thank you for your cooperation!

1. The service scope of this site includes: _____ streets and _____ communities; the service coverage of permanent population includes: _____ people (the permanent population refers to the population who has lived in the community for six months or more)
2. The service coverage radius of this site is: _____
3. Within 5 kilometers; ② 5-10 kilometers; ③ more than 10 kilometers
4. The total staff of this site: _____

Among them: Doctors: _____, Number of men: _____

Among them: Nurses: _____, Number of men: _____

Other personnel (drivers and stretchers, etc.): _____ , number of men: _____

1. Among the doctors on this site:

Graduate: _____

Undergraduate: _____

Junior College: _____

Technical Secondary: _____

High School: _____

1. Among the nurses on this site:

Graduate: _____

Undergraduate: _____

Junior College: _____

Technical Secondary: _____

High School: _____

1. Among the titles of doctors on this site:

Senior Level: _____

Vice Senior Level: _____

Intermediate Level: _____

Teacher/Assistant Level: _____

Primary Level: _____

1. Among the titles of nurses on this site:

Senior Level: _____

Vice Senior Level: _____

Intermediate Level: _____

Teacher/Assistant Level: _____

Primary Level: _____

1. Among the doctors on this site:

Under 25: _____

25～34 years old: _____

35～44 years old: _____

45 years old and above: _____

1. Among the doctors on this site:

Under 25: _____

25～34 years old: _____

35～44 years old: _____

1. years old and above: _____
2. There are _____ ambulances on this site; the number of ambulances equipped with electrocardiograph and simple ventilator is _____
3. Considering the number of people served by this site, do you think the medical staff on this site can meet the demand?
4. Yes; ② No; ③ Not sure
5. Considering the population served by this site, do you think the ambulances equipped on this site can meet the demand?
6. Yes; ② No; ③ Not sure
