# Supplementary material for: Quantity and Quality of Healthcare Professionals, Transfer Delay and In-hospital Mortality Among ST-Segment Elevation Myocardial Infarction: A Mixed-Method Cross-Sectional Study of 89 Emergency Medical Stations in China
Source: Front Public Health. 2022 Jan 24;9:812355. doi: 10.3389/fpubh.2021.812355 (PMC8818716; doi:10.3389/fpubh.2021.812355)
Supplement: Supplementary file 3 [file Table_3.DOCX]

**Interview Guide 1 Director**

Name of interviewee： Gender： Occupation：

Institution： Date： / /

Brief description of the interview environment process：

Investigator： Recorder：

1. What is your management of emergency medical dispatch like?
2. Do you think you have any difficulties in managing staff?
3. What is personnel incentive mechanism in your emergency medical station, as well as the results or problems of these practices, and how to improve them?
4. How do you feel about the motivation and motivation of your employees? If their work is positive, what motivates them? If they are passive, what are the factors that cause them? How can it be improved? Do you think there are any ideas, ideas or practices to mobilize the enthusiasm of the staff?
5. What measures have you taken to attract and retain talent? What are the problems and how can they be improved?
6. What measures have you taken to encourage employees to take part in training or academic education? What are the problems or difficulties in personnel training? How can it be improved?
7. What do you think is the reason for the lack of healthcare professionals?

**Interview Guide 2 Physicians and Nurses**

Name of interviewee： Gender： Occupation：

Institution： Date： / /

Brief description of the interview environment process：

Investigator： Recorder：

1. Are you passionate about your job?

2. How satisfied is your job? If you are more passive, what are the factors that cause it? What do you think your institution should do to improve your work motivation?

3. What do you think of your current incentive mechanism? Are these incentive mechanisms working for you? What do you think are the problems? How can they be improved?

4. What kind of incentive mechanism do you think works best for you?

5. What do you think are the problems or difficulties in personnel training in your institution? How can they be improved?
